# Blended Delivery of Imagery Rescripting for Childhood PTSD: A Case Study During the COVID-19 Pandemic

**DOI:** 10.32872/cpe.7815

**Published:** 2022-09-30

**Authors:** Nathan Bachrach, Sanja Giesen, Arnoud Arntz

**Affiliations:** 1 Department of Medical and Clinical Psychology, Tilburg University, Tilburg, The Netherlands; 2 GGZ Oost Brabant, Boekel, The Netherlands; 3 RINO Zuid, Eindhoven, The Netherlands; 4 Department of Clinical Psychology, University of Amsterdam, Amsterdam, The Netherlands; Philipps-University of Marburg, Marburg, Germany

**Keywords:** imagery rescripting, PTSD, telehealth, childhood trauma-related PTSD

## Abstract

**Background:**

Despite the growing evidence that trauma-focused treatments can be applied as first-line approaches for individuals with childhood trauma-related PTSD (Ch-PTSD), many therapists are still reluctant to provide trauma-focused treatments as a first-choice intervention for individuals with Ch-PTSD, especially by telehealth. The current manuscript will therefore give an overview of the evidence for the effectiveness of trauma-focused therapies for individuals with Ch-PTSD, the delivery of trauma-focused treatments via telehealth, and a case example on how a specific form of trauma focused therapy: Imagery Rescripting (ImRs) can be applied by telehealth.

**Method:**

This article presents a clinical illustration of a blended telehealth trajectory of imagery rescripting (ImRs) Ch-PTSD delivered during the COVID-19 pandemic.

**Results:**

The presented case shows that ImRs can be safely and effectively performed by telehealth for ch-PTSD, no stabilization phase was needed and only seven sessions were needed to drastically reduce Ch-PTSD and depressive symptoms, and to increase quality of life.

**Conclusion:**

This case report shows the effectiveness of ImRs by telehealth for Ch-PTSD, which gives hope and additional possibilities to reach out to patients with ch-PTDS. Telehealth treatment might have some of advantages for specific patients, especially, but certainly not only, during the pandemic.

## Highlights

- ImRs is an effective and highly acceptable procedure for both patients as therapists and seems a very good option for treating Ch-PTSD effectively.
- In the presented case only seven sessions were needed to reduce Ch-PTSD and depressive symptoms drastically, and increase quality of life.
- The delivery of ImRs by telehealth did not have a negative impact on the effectiveness, quality and patient satisfaction; which is in line with systematic reviews on effectiveness of psychological telehealth treatments for PTSD (not ImRs and not specifically Ch-PTSD).

Meta-analytic reviews and practice guidelines recommend Trauma-Focused Cognitive Behavior Therapy (TF-CBT) and Eye Movement Desensitization and Reprocessing (EMDR) as first-line treatments for PTSD (Lewis et al., 2020). Despite the growing evidence that trauma-focused treatments can be applied as first-line approaches for individuals with childhood trauma-related PTSD (Ch-PTSD), many therapists are still reluctant to provide trauma-focused treatments as a first-choice intervention for individuals with Ch-PTSD, especially by telehealth (Wild et al., 2020). The current manuscript will therefore give an overview of the evidence for the effectiveness of trauma-focused therapies for individuals with Ch-PTSD, the delivery of trauma-focused treatments via telehealth, and a case example on how a specific form of trauma focused therapy: Imagery Rescripting (ImRs) can be applied by telehealth.

Individuals with Ch-PTSD are characterized by more complex PTSD symptoms, such as emotional regulation problems, interpersonal difficulties and impaired self-concept (Ehring et al., 2014; Messman-Moore & Bhuptani, 2017). There is a limited number of studies investigating trauma-focused treatment among Ch-PTSD patients (Ehring et al., 2014). A meta-analysis of psychological treatments for Ch-PTSD (Ehring et al., 2014) found evidence that patients with Ch-PTSD can be treated safely with trauma-focused therapies, and that these treatments are effective (moderate to high effect sizes) in reducing PTSD symptoms as well as related symptoms, such as depression, anxiety and dissociation. Furthermore, recent randomized controlled studies show that direct applications of trauma-focused therapies such as prolonged exposure, EMDR, and Imagery Rescripting are very effective and can be performed safely with Ch-PTSD patients (Boterhoven de Haan et al., 2020; Oprel et al., 2021). These studies found large effect sizes for reducing PTSD symptoms as well as other symptoms such as depression, dissociation and trauma related cognitions with trauma-focused treatments in Ch-PTSD patients, with notably low dropout rates (7%) for EMDR and ImRs compared to Prolonged Exposure and Intensified Prolonged Exposure (27% and 29%) and low rates of serious adverse events (Boterhoven de Haan et al., 2020; Oprel et al., 2021).

ImRs as a stand-alone treatment for Ch-PTSD has been studied far less compared to other first line PTSD treatments such as EMDR, Prolonged Exposure, Cognitive Processing Therapy and TF-CBT. Recent findings show that ImRs is a very effective procedure and is highly acceptable for both patients as therapists (Boterhoven de Haan et al., 2020; Boterhoven de Haan et al., 2021; Morina et al., 2017; Raabe et al., 2015). Very large treatment effects on the Clinician Administered PTSD Scale for DSM-5 between baseline and one-year follow-up (i.e., pre-post *d* = 2.26 for ImRs and *d* = 1.88 for EMDR) were found in a recent RCT in which EMDR was compared to ImRs. Moreover, the drop-out rates were low, at 7.7%, suggesting that the treatments were well tolerated by participants (Boterhoven de Haan et al., 2020). No differences in effectiveness and dropout between EMDR and ImRs for Ch-PTSD, were found. However, ImRs was superior for those with comorbid depression, which is highly prevalent in PTSD patients (70% in the IREM sample) (Assmann et al., 2021). To date, cost effectiveness studies in ImRs have not yet been performed. ImRs might be potentially more cost-effective than EMDR, because of lower training costs and shorter sessions (60 vs 90 minutes). It is also still not clear how session frequency impacts the effectiveness of PTSD treatments, whether treatment type moderates the frequency effect, and which treatment type and frequency works best for which patient (Wibbelink et al., 2021). ImRs uses a different method compared to prolonged exposure and EMDR, therefore, ImRs might additionally work for patients who do not benefit from other PTSD treatments. Research shows that ImRs compared to prolonged exposure leads to less dropout (Arntz et al., 2007) is experienced as less distressing (Siegesleitner et al., 2019) and is more effective regarding anger control, hostility and guilt. ImRs might therefore be indicated especially for a specific group of patients who experience difficulties in these areas and PTSD patients with comorbid depression (Assmann et al., 2021; Bosch & Arntz, 2021).

In ImRs for PTSD, patients are asked to vividly recall a traumatic experience whereafter patients are asked to imagine that an intervention takes place that changes the course of the original memory into an image in which the needs of the patient are fulfilled (Arntz, 2012; Arntz & Weertman, 1999). In ImRs several therapeutic steps are used to modify the content of traumatic memories into new positive images in order to change the meaning of the trauma memory representation, by adding new and corrective information about the meaning of the event. ImRs is thought to reevaluate unconditioned stimuli and thereby reduce conditioned stimuli-elicited affects (Arntz, 2012). This is done by adding new information into the memory representation of the unconditioned stimuli; by for instance adding information on the needs of little children and taking care of the patients’ needs in the traumatic event. ImRs for PTSD is performed in phases. In the first phase, which usually has a duration of six sessions, patients are asked to close their eyes and imagine a concrete negative traumatic experience as vividly as possible, until enough emotional arousal is achieved usually around a specific traumatic moment in the memory representation. Prolonged exposure to the most traumatic aspect of the memory is not necessary, the therapist enters the image when arousal levels are still manageable for the patient. The therapist rescripts the image by establishing safety for the child, and in the following steps, further needs of the child are taken care of, and the child's emotions are validated. The perpetrator is confronted and hold accountable for their actions and responsibility and, if necessary, helped to do better in the future or to punished and/or eliminated so he/she cannot cause any harm. In the second phase of treatment, usually after 6 sessions, after trauma-memory activation (from the child perspective), ImRs is performed in three steps (1) patients are asked to imagine the image as an adult in order to experience what they feel, think and are inclined to do from their present adult perspective. (2) Thereafter they are stimulated to intervene in the image and do whatever they think is needed for their own little child. (3) Patients are subsequently asked to experience the interventions by their adult self again from the perspective of the child in order to experience how it feels when needs are fulfilled (Arntz & Weertman, 1999). A recent study investigated the perspectives of patients and therapists regarding the elements of change in IMRS. Patients mention, caring for the child by the therapist when the therapist rescripts the traumatic event, speaking up to the perpetrator, the positive connection they had with the therapist and the encouragement they received from him or her as important elements of change (Bosch & Arntz, 2021).

Delivering ImRs by telehealth (e.g., delivering psychological therapy remotely via video teleconferencing) to patients with PTSD poses challenges to both therapist and patients (Paulik et al., 2021). The need for remote delivery of psychological treatments increased drastically due to the COVID-19 pandemic, because of closure of outpatient facilities, travel restrictions, and home confinement. Up to date, the study of delivery of ImRs via telehealth has been limited to a few cases (Paulik et al., 2021). However, several systematic reviews on effectiveness of psychological telehealth treatments for various disorders including PTSD (not ImRs and not specifically Ch-PTSD) have been performed, which in general show that the effectiveness, drop-out rates, quality and patient satisfaction, is comparable to face-to- face therapies (Berryhill et al., 2019; Bolton & Dorstyn, 2015; Finkelstein et al., 2006; Simpson, 2009; Sunjaya et al., 2020; Varker et al., 2019). Despite this ample evidence to support the use of telehealth therapy for mental health conditions, therapists and patients however may be hesitant to perform telehealth therapies targeting memories of traumatic experiences. Paulik and colleagues (2021) describe key clinical considerations and recommendations for delivering ImRs by telehealth: the importance to consider the context (living condition, level of privacy during therapy, levels of COVID-19 restrictions, voluntariness of choice for telehealth) perceived and real safety (being physically safe and having a safe place to perform ImRs); practical (travelling time, preparation structure of sessions, camera position, exhaustion levels of therapist, quiet environment) and technological issues (stable connection, type of device) therapeutic alliance (reduced level of eye contact, more difficult observation of body language); depth of emotional processing (stimulating visualization and emotionally connect to the image); and dissociation (strategies to stop dissociation). ImRs might be more easily adapted to telehealth delivery than other trauma-focused methods such as EMDR because ImRs does not require dual stimulation tasks, and during rescripting patients have their eyes closed and are not focused on the therapist. ImRs also does not require the provision of materials in the sessions, such as handouts for completion of homework (Paulik et al., 2021). In the following a case illustration is given of the application ImRs protocol by telehealth.

## Case Illustration

### Presenting Problem and Client Description

The case report is presented with permission of the patient, for privacy reasons several changes were made to the report (e.g. names, dates). Larry is a 42-year-old divorced, unemployed man, who was referred by the Assertive Community Treatment team (ACT) to the trauma department of a mental health care center in the Netherlands for treatment of Ch-PTSD. ACT is a service-delivery treatment model that provides comprehensive, locally based treatment to people with serious and persistent mental illnesses (Drukker et al., 2011). During the assessment phase the following DSM-5 classification was made based on the following semi-structured clinical interviews: SCID-5-P (First et al., 2015); SCID-5-CV (First et al., 2016) and CAPS-5 (Weathers et al., 2018): antisocial personality disorder with schizoid- and borderline personality traits; depression, ADHD and chronic childhood Ch-PTSD. He suffers from low self-esteem, difficulties in aggression regulation, and difficulties with maintaining intimate relationships. He feels detached from others, is hyperalert, worries a lot and can be impulsive, experiences nightmares and sleeping problems. Larry functioned on the fringes of society for several years, but has recently found a volunteer job and now lives independently. He has a limited social network because of his distrust of others.

Larry grew up in a family in which he did not feel safe and connected. Larry was the middle child of three. Larry has few memories of his childhood and mentions that he was a hyperactive and difficult child. He felt unwanted as a child and had an emotionally detached father who worked a lot and a gentle mother who was a housewife. She died in a car accident caused by a drunken driver when Larry was 6 years old. Larry was not allowed to attend the funeral because his father did not let him attend it. After his mother passed away, his brother, grandparents, and friends looked after Larry when Larry’s dad attended work. Larry was sexually abused by the father of a befriended family, who baby-sat Larry, from his seventh till tenth year of age. At school Larry experienced concentration and behavioral problems and regularly got into fights. There was very little support and attention for him at home. He went to technical secondary vocational education. He quit school after getting beaten up by a group of boys at age sixteen. He met his wife at age eighteen, they married after she got pregnant. Larry once forced his wife to have sex after which she filed a divorce. She left him with their child, which was three years old at that time. After the divorce, Larry did not see his daughter anymore. Larry held numerous jobs and often experienced conflicts at work. He got addicted to gambling and into serious debts, he lost his house and lived on the street for three years. During this stressful period, he experienced several psychotic episodes; the first around age of thirty-two. Because of the psychotic experiences Larry sought mental help and his general practitioner referred him to a ACT team. The ACT team helped him to reduce psychotic problems, depressive complaints and helped him to live independently again. He found volunteer work and restored contact with one brother and one friend. His psychotic symptoms were resolved and he succeeded in living independently again. He now lives a tranquil and isolated life, with which he seemed satisfied. Larry drank about five beers a day and smoked weed occasionally; he received anti-psychotic-, anti-depressants-, anti-ADHD- and sleep medication. He was referred for trauma therapy by the ACT team and was offered to take part in the IREM-freq study.

The IREM-freq study design is registered in NTR7153 and approved by the ethics committee of the University Amsterdam. The design manuscript of the study was submitted recently (Wibbelink et al., 2021). Larry was randomly allocated to the two times a week ImRs condition, 90 minutes per session with a maximum of 12 sessions. In the IREM-freq ImRs protocol the therapist rescripts the traumatic situation in the first six sessions, from the seventh session till twelfth session the patient as his current self-rescripts the traumatic event (Boterhoven de Haan et al., 2020). Due to the pandemic, the face-to-face sessions had to be stopped, and treatment was continued online. Because of methodological considerations, the study participants that could not be treated vis-à-vis were excluded from the IREM-freq study (see Wibbelink et al., 2021, and trial registration). Therefore, the case of Larry could be separately presented. Larry was a friendly, quiet, reserved but cooperative man who made the impression to be at ease living an isolated life. The therapist felt sympathy and empathy for him. Larry did not show any aggressiveness to the therapist, nor did he evoke any negative or intense countertransference emotions.

### Course of Treatment

The first two sessions were delivered face-to-face, after which the COVID-pandemic led to delivering the treatment online. In the first session trauma processing does not takes place. In this session the therapist got acquainted with Larry and explained the rational of ImRs. The therapist and Larry made a list of traumas that Larry wanted to address. This list included trauma’s that contributed to the PTSD diagnosis as well as traumas that did not qualify for the A-criterion of PTSD in the DSM-5 definition of PTSD. The list is considered to be flexible, the patient can add trauma’s during treatment and/or can change the order in which traumas are addressed. In the first session a trial ImRs intervention with a mildly negative memory, preferable before age of 12, is provided to let patients become familiar with ImRs. In Larry’s case this was getting beaten up at school.

Larry’s list of traumas included the following themes:

- Sexual abuse at age seven till ten years of age
- Death of his mother at the age of six
- Getting beaten up by a group of boys at age 16
- Being threatened by a motorcycle gang at age 40
- Aggressive behavior against and sexual abuse of his ex-wife at age 22

In the second session, the first active ImRs session, the loss of his mother due to a car accident was processed. Larry was not allowed to see his mother after the accident and to attend the funeral. Larry therefore was not able to properly take part in his mother’s farewell. His family members didn’t talk about her death after the funeral.

Therapist Please close your eyes Larry, I would like you to speak in the present tense and the I-form as if the situation which we will process is happening right now. Please go back to the situation where your mother died. Where are you? What is happening?

Larry I see my mom; she is crushed in the car (Crying). I’m overwhelmed and feel sad.

Therapist What do you need?

Larry I’m so lonely. Somebody should comfort me.

Therapist I’m here. Oh Larry, this must be so sad for you. It is okay to cry, losing your mommy is a great loss. I’ll take care of you. Let me comfort you. I’ll put my arms around you. Is there anything else you need?

Larry I feel calmer now.

Therapist And the drunk driver, is something needed towards him?

Larry Yes! He gets away with it. He should be punished.

Therapist Okay, I’m still there, I’ll confront this driver. How can you be so irresponsible? Do you realize what you have done? You just killed a mother of this friendly little boy who needs his mother. And how dare you to just leave the scene of the accident and just drive through. This is a crime; you belong in jail! Police officer please incarcerate this man.

Larry I see them taking him away.

Therapist What would you like to happen now?

Larry I want to see my mom and tell her that I love her. I want to say goodbye.

In the rescripting Larry felt very lonely and in need of support and comfort. He felt relieved to get comforted in the rescripting *and* experienced a reduction of feelings of revenge towards the drunken driver.

Because of the COVID-19 pandemic and the government restrictions of the lockdown Larry was not able to attend physical appointments. Larry was therefore asked if he would like to continue the ImRs by telehealth, to which he agreed. In the following session the use of telehealth by secured video call was set up, because the sound of the video call was of poor quality the audio of the videocall was delivered by phone.

The third ImRs session was performed via telehealth. Notably Larry was very much at ease at his own home, he was drinking coffee, smoked cigarettes and spontaneously interrupted the sessions by going to the toilet and was distracted by his cat who walked on his keyboard. These behaviors are not uncommon when delivering therapy by telehealth (Paulik et al., 2021). Practical agreements such as quiet environment without distractions should be made, preferably in advance, in order to perform ImRs successfully by telehealth. The therapist and Larry therefore discussed how Larry could best profit from the telehealth sessions. They agreed on Larry attending telehealth sessions similar to the face-to-face sessions (e.g. no distraction, drinks and toilet visits, the ImRs procedure could thereafter proceed in exactly the similar manner as to face-to-face ImRs. After these ground rules were set, therapist and Larry carried on with the ImRs procedure and succeeded to process the most important index trauma the sexual abuse. The (index) trauma that they worked on was memory of the first time that the sexual abuse took place. This was a situation in a car in which he had to perform oral sex The abuse always took place in this car; therefore, this situation was exemplary of the majority of the sexually abuse experiences. In the rescripting Larry felt very anxious and in need of protection.

Therapist I step into the image, do you see me? I take you out of the car immediately, come and stay behind me Larry. I lock the car, he cannot get out anymore. What are you doing? You are damaging Larry, that is very bad and mean. I brought police officers with me. Arrest this man! He is abusing Larry. Incarcerate this filthy man! What do you see Larry?

Larry I see him being taken away.

Therapist What else do you need?

Larry I feel shaky and anxious. What happens when they let him go?

Therapist I’ll tell the police to lock him up forever. He will not be able to hurt you anymore. You are safe now Larry. Is there anything else you need?

Larry I feel lonely and sad.

Delivering safety to Larry was followed by condolement. Larry wished his dad would comfort and help him. In the rescripting it became clear that Larry’s father was not able to comfort Larry, he neglected Larry. The therapist therefore confronted Larry’s dad that it is his task as a parent to take care for Larry and provided safety and comfort to little Larry. Thereafter, therapist continued rescripting by giving comfort to little Larry. Little Larry was explained that he was not guilty of the abuse, but the perpetrator was, and that the man who abused him misused the vulnerability of Larry and should be punished for his actions. The therapist reassures little Larry that if needed the therapist would be there for Larry, *therapist: every time you’ll need me, I’ll be there for you.* At the end of the rescripting Larry wants to play soccer with a friend. Larry feels happier and less guilty at this point.

In the 4th session (second telehealth session) Larry wanted to address his own sexually aggressive behavior towards his ex-wife, which led to a divorce and loss of contact with his child. The hotspot in this situation was his sexually behavior in their bedroom.

Therapist Okay Larry, I’m here. What do you need right now?

Larry I feel so bad, I want to stop myself. Because Larry explicitly wanted to take action himself, the therapist decided to violate the ImRs protocol by letting adult Larry rescript the situation (fourth session instead of seventh session), assuming that it would be more powerful and effective when Larry would address his own aggressive behavior.

Therapist What does adult Larry want tell to the twenty-two-year-old Larry, go ahead tell him.

Larry You fool, stop immediately. You should never do this; this is so wrong. Get out, you are destroying your life and that of your family.

Therapist How does the twenty-two-year-old Larry react?

Larry He startles, he’s ashamed so badly (crying).

Therapist What else would you like to do?

Larry I want to tell my wife I’m sorry.

Therapist Okay, tell her as current Larry what you want to say, go ahead.

In the rescripting, current Larry acknowledges to his ex-wife that he has a deep remorse.

Therapist Is there anything else you feel like doing?

Larry I want to tell my daughter how sorry I am. That I’ve been wrong. I don’t want to take the father role in her life. But I want her to know I miss her.

Therapist Go ahead Larry.

Current Larry apologizes to his current daughter in the rescripting. After that he feels the need for comfort because of all his losses, the therapist offers this to him in the image by giving him a hug and by validating his feelings and speaking out comforting words. He feels relieved afterwards.

In the 5th session (third via telehealth) Larry addresses a situation in which he was beaten up by a group of boys. The sudden confrontation appeared to be the most traumatic point of the memory of this situation. Little Larry felt in need of safety and wanted to escape the boys.

Therapist I want you to know that it is not your fault. This must have been very scary for you. They should be ashamed that they’re threatening you and beat you up while they’re with so many. How do you feel now? What do you need?

Larry I’m very angry. They should be punished. They should experience the same as what they did to me.

Therapist You guys are so bloody mean; you should feel ashamed of yourselves. I’ll hit you and kick you wherever I can. If you’ll do this again, you’ll meet me once again. How do they react? Did I punish them enough or is more needed?

Larry I don’t know. It feels bad to see them beaten up. I rather have the police take them into custody, let them be scared.

Therapist Very well, we rewind the film.

Therapist You have no right to be so cruel to this boy. I have brought police officers with me. Police officers take care of this scum, make sure they’ll never harm Larry again and to inform their parents about their gratuitous violence.

Once Larry felt safe he felt that the boys needed to be punished for their deeds and he wanted to be sure that they would never harm him again. The therapist informed the police who took the boys into custody.

Therapist Does it feel okay for you now, or are you in need of something else?

Larry It is okay, I feel calm now.

Therapist Shall we do something nice?

Larry Let’s play soccer.

Therapist Let’s go to the square and have some fun. Feel the sun, smell the grass. Enjoy playing soccer for a while…… open your eyes and return to the here and now.

Larry felt relieved and at ease after the ImRs.

In the 6th session Larry chose a situation in which he was threatened by a motorcycle gang whereby he felt very unsafe for several days. At the start of the threats, he didn’t dare to go to sleep for days which resulted in sleep deprivation and subsequently a psychotic episode. The most traumatic moment of the memory in this situation was the moment he was told that he was dating a former girlfriend of a member of the motor cycle gang and he realizes he is in trouble. In the rescripting Larry feels anxious and is in need of safety. Therapist rescripts the situation by rescuing Larry by taking him out of the situation. After Larry is safe, he wants to be sure they’ll never harm him again. The motor gang is incarcerated by the police and they get locked up. Larry feels relieved and at ease after the rescripting.

From the 7th session (fifth telehealth session), following the protocol, the patient rescripts the traumatic event as his current self and therapist coaches the adult-self when necessary, to do what is needed. At the start of the session Larry shares that he no longer feels anxious when he hears the sounds of motor bikes. In this session Larry wants to address the sexual abuse because it is still bothering him, the same traumatic situation in which Larry got abused again was processed for a second time. At first Larry was asked to step into the situation as little Larry. The most traumatic aspect of the memory of the situation was the moment at which the sexual abuse was going to take place.

Therapist What do you need right now?

Larry I want to get out of the car, I want to get away.

Therapist Okay, keep your eyes closed and step into the situation as adult Larry. What is happening? What do you see?

Larry I see little Larry, he is so scared. It makes me angry.

Therapist What would you like to do right now?

Larry I want to beat the man up and to have the police lock him up forever. He is not allowed to abuse little Larry.

Therapist Okay. Go ahead, do what you want to do.

Adult Larry confronts the abuser.

Larry I tell him he is not worth living, that he is really disgusting and that everybody should know what he has done, you’re a fucking loser. I beat the hell out of him.

Therapist How does little Larry react?

Larry He feels that justice is done. He is peaceful now.

Therapist Larry what inclination do you have now?

Larry I want to tell little Larry that he can’t help it, he is innocent and a good boy. I am always there for him.

Therapist Very well, just say that directly to little Larry.

Larry [speaks directly to little Larry]

Therapist Is there anything else that’s needs to be done?

Larry No, it is okay now.

Therapist What about your father, does he need to know?

Larry Maybe. I don’t know, we can try.

Therapist Let’s go to your father, what do you want to tell him?

Larry I tell him what happened.

Therapist How does your father react?

Larry He startles. He feels uncomfortable. He doesn’t know how to react.

Therapist Is there anything else you want to tell him?

Larry You should have been more careful, and looked after little Larry; he needs you to be there for him.

Therapist How does little Larry react?

Larry He is sad. He needs a hug. My father is never going to give him that.

Therapist Is there anything you can do for little Larry?

Larry I’ll hug him till he is calm.

When adult Larry feels satisfied the therapist tells Larry: *Ok, keep your eyes shut. Go back to the situation, but now as little Larry and start the film from beginning and tell me what happens when adult Larry intervenes*? In the end the therapist asks little Larry*: What would you want to happen right now?*

Little Larry It feels awkward that my father doesn’t know how to react. I want adult Larry to tell it is not my fault.

Therapist Go ahead, ask adult Larry what you need. What does adult Larry do?

Little Larry He tells me I’m a good boy, that it is not my fault that father is clumsy. A father shouldn’t be that neglective but should be giving his son attention and see his need for comfort after having lost his mother and after what he went through during the abuse. He tells me that father is not capable of giving that to me. He gives me a hug.

After which they leave the house to play soccer together, they have fun and the little Larry feels relaxed.

In the eight session (sixth telehealth session) Larry states that he is no longer experiencing nightmares and flashbacks and feels that he has progressed enormously in treatment; he does not have any situations anymore which he wants to address. Larry also feels at ease that there currently is no contact with his daughter and grandchildren, he feels resignation about this situation. Larry wished that he received ImRs much earlier in his life. After PTSD treatment, Larry was able to reduce his antidepressant (90%) and sleep medication (50%). Larry was inclined to stop with the ACT treatment which he received for several years.

### Therapy Outcome and Prognosis

Larry was considered an early completer because he only needed seven out of twelve sessions. Therapy outcome and prognosis for Larry, were very good. The follow-up assessment of self-reported and clinician administered PTSD symptoms, quality of life, general psychiatric symptoms and trauma related cognitions about self and others showed dramatic, significant and clinical improvements (see Table 1). The prognosis of Larry is expected to be very good based on the follow up results after 1 year.

**Table 1:** Results on Outcome Measures at Baseline, After Completion of ImRs and Follow up

Measure	Baseline	After completion of ImRs	1 year follow up
Clinician Administered PTSD Scale for DSM-5 (Weathers et al., 2018)	33	9	3
PCL-5 index trauma (Weathers et al., 2013)	51	10	15
PCL-5 other traumatic events	51	9	15
WHODAS 2.0 (Üstün et al., 2010)	47.92	16.67	12.50
Symptom Check List-90 Hostility (Derogatis & Unger, 2010)	6	6	6
PTCI (Foa et al., 1999)			
Negative cognitions about self	4.48	2.14	1.86
Negative cognitions about world	5.57	2.86	1.71
Self-blame	5.40	2.40	4.40
EuroQol EQ-5D-5L Quality of life VAS (Busschbach et al., 2016). Average of general Dutch population age group 40-49 = .85, *SD* = .20)	0.43	0.77	0.85
Beck Depression Inventory BDI-II (Beck et al., 1996).	28	6	7
Happiness (Abdel-Khalek, 2006).	Fairly unhappy	Fairly happy	Entirely happy

## Discussion

Larry’s case is unfortunately exemplary for patients with Ch-PTSD in which underdiagnosis and undertreatment is common. PTSD is often not diagnosed; only 2% to 11% of the patients with PTSD actually have their diagnosis noted in the medical record in primary care and 18-35% in mental health care centers (Kantor et al., 2017; Meltzer et al., 2012). Unfortunately, less than half of the patients with PTSD diagnosed, or even fewer, actually receive treatment for PTSD (Kantor et al., 2017; Meltzer et al., 2012). This creates a major risk for escalation of clinical disorders (such as psychosis in Larry’s case), chronicity and long treatments, poor quality of life and high societal costs. Early detection and treatment of PTSD urgently is needed in order to counter these negative effects. ImRs has proven to be an effective and highly acceptable procedure for both patients as therapists and seems a very good option for treating Ch-PTSD effectively (Boterhoven de Haan et al., 2020; Morina et al., 2017; Raabe et al., 2015). In Larry’s case, which at forehand seemed to be a very complex case, only seven sessions were needed to reduce PTSD and depressive symptoms drastically, and increase his quality of life. By just following the treatment protocol, the application of ImRs could be performed by telehealth in a regular manner in quiet a complex case. This is in line with research previous findings which show that trauma focused therapy (e.g., EMDR and imaginal exposure) is effective, safe, and feasible in patients with PTSD and complex symptoms such as severe psychotic disorder (van den Berg et al., 2015). The delivery of ImRs by telehealth did not seem to have a negative impact on the effectiveness, quality and patient satisfaction; which is in line with systematic reviews on effectiveness of psychological telehealth treatments for PTSD (not ImRs and not specifically Ch-PTSD) (Berryhill et al., 2019; Bolton & Dorstyn, 2015; Finkelstein et al., 2006; Simpson, 2009; Sunjaya et al., 2020; Varker et al., 2019). In Larry’s case the delivery of ImRs by telehealth proceeded similar to face-to-face sessions. It might have helped that the initial start of the trajectory was face-to-face due to which patient and therapist got acquainted before switching to telehealth. Larry’s context might be favorably to telehealth, he had had a good internet connection, lived by himself –privacy was guaranteed - and was motivated to continue treatment by telehealth. It however might be helpful if basic agreements on how ImRs by telehealth is delivered and in which manner patients can take care for privacy, quiet environment, good stable internet connection, focus during sessions and how to deal with possible dissociation, were discussed in advance (Paulik et al., 2021). It is important to investigate the effectiveness of delivering ImRs by telehealth in an adequately designed and powered study. Furthermore, it is unlikely that telehealth is applicable to all patients; some patients might respond better to face-to-face ImRs compared to ImRs delivered by telehealth. It is important to investigate patient and context characteristics in order to improve treatment selection. It is important to note that application of telehealth and effectivity of telehealth might also depend on therapists' attitudes towards telehealth applications. Therapists might be reluctant to perform PTSD treatments online, which might interfere with outcomes and extensive application of telehealth. However, several systematic reviews on effectiveness of psychological telehealth treatments for various disorders including PTSD show that the effectiveness, drop-out rates, quality and patient satisfaction, is comparable to face-to-face therapies (Berryhill et al., 2019; Bolton & Dorstyn, 2015; Finkelstein et al., 2006; Simpson, 2009; Sunjaya et al., 2020; Varker, Brand et al., 2019). Which might indicate that this reluctance and attitudes towards telehealth might not be justified. In conclusion, Larry’s case illustrates that ImRs can be safely and effectively performed by telehealth for ch-PTSD, no stabilization phase was needed and only seven sessions were needed to drastically reduce Ch-PTSD and depressive symptoms, and to increase quality of life. This gives hope and additional possibilities to reach out to patients with ch-PTDS due to the fact that telehealth might have some of advantages for patients, especially, but certainly not only, during the pandemic. This patient group is so often undertreated for their PTSD, this case report shows that the reluctance for direct PTSD treatment through telehealth is not rightfully.

## References

[r1] Abdel-Khalek, A. M. (2006). Measuring happiness with a single-item scale. Social Behavior and Personality, 34(2), 139–150. 10.2224/sbp.2006.34.2.139

[r2] Arntz, A. (2012). Imagery rescripting as a therapeutic technique: Review of clinical trials, basic studies, and research agenda. Journal of Experimental Psychopathology, 3(2), 189–208. 10.5127/jep.024211

[r3] Arntz, A., Tiesema, M., & Kindt, M. (2007). Treatment of PTSD: A comparison of imaginal exposure with and without imagery rescripting. Journal of Behavior Therapy and Experimental Psychiatry, 38(4), 345–370. 10.1016/j.jbtep.2007.10.006 18005935

[r4] Arntz, A., & Weertman, A. (1999). Treatment of childhood memories: Theory and practice. Behaviour Research and Therapy, 37(8), 715–740. 10.1016/S0005-7967(98)00173-9 10452174

[r5] Assmann, N., Fassbinder, E., Schaich, A., Lee, C. W., Boterhoven de Haan, K., Rijkeboer, M., & Arntz, A. (2021). Differential effects of comorbid psychiatric disorders on treatment outcome in posttraumatic stress disorder from childhood trauma. Journal of Clinical Medicine, 10(16), 3708. 10.3390/jcm10163708 34442005 PMC8397108

[r6] Beck, A. T., Steer, R. A., & Brown, G. K. (1996). *Manual for the Beck Depression Inventory-II.* San Antonio, TX, USA: Psychological Corporation.

[r7] Berryhill, M. B., Halli-Tierney, A., Culmer, N., Williams, N., Betancourt, A., King, M., & Ruggles, H. (2019). Videoconferencing psychological therapy and anxiety: A systematic review. Family Practice, 36(1), 53–63. 10.1093/fampra/cmy072 30188992

[r8] Bolton, A. J., & Dorstyn, D. S. (2015). Telepsychology for posttraumatic stress disorder: A systematic review. Journal of Telemedicine and Telecare, 21(5), 254–267. 10.1177/1357633X15571996 25712113

[r9] Bosch, M., & Arntz, A. (2021). Imagery rescripting for patients with posttraumatic stress disorder: A qualitative study of patients’ and therapists’ perspectives about the elements of change. Cognitive and Behavioral Practice. Advance online publication. 10.1016/j.cbpra.2021.08.001

[r10] Boterhoven de Haan, K. L., Lee, C. W., Correia, H., Menninga, S., Fassbinder, E., Köehne, S., & Arntz, A. (2021). Patient and therapist perspectives on treatment for adults with PTSD from childhood trauma. Journal of Clinical Medicine, 10(5), 954. 10.3390/jcm10050954 33804440 PMC7957589

[r11] Boterhoven de Haan, K. L., Lee, C. W., Fassbinder, E., van Es, S. M., Menninga, S., Meewisse, M.-L., Rijkeboer, M., Kousemaker, M., & Arntz, A. (2020). Imagery rescripting and eye movement desensitisation and reprocessing as treatment for adults with post-traumatic stress disorder from childhood trauma: Randomised clinical trial. The British Journal of Psychiatry, 217(5), 609–615. 10.1192/bjp.2020.158 32892758

[r12] Busschbach, J. et al. (2016). *QALY en kwaliteit van leven metingen.* Diemen ZiNL.

[r13] Derogatis, L. R., & Unger, R. (2010). Symptom checklist‐90‐revised. In *The Corsini Encyclopedia of Psychology*, 1-2. 10.1002/9780470479216.corpsy0970

[r14] Drukker, M., van Os, J., Sytema, S., Driessen, G., Visser, E., & Delespaul, P. (2011). Function assertive community treatment (FACT) and psychiatric service use in patients diagnosed with severe mental illness. Epidemiology and Psychiatric Sciences, 20(3), 273–278. 10.1017/S2045796011000369 21922970

[r15] Ehring, T., Welboren, R., Morina, N., Wicherts, J. M., Freitag, J., & Emmelkamp, P. M. (2014). Meta-analysis of psychological treatments for posttraumatic stress disorder in adult survivors of childhood abuse. Clinical Psychology Review, 34(8), 645–657. 10.1016/j.cpr.2014.10.004 25455628

[r16] Finkelstein, S. M., Speedie, S. M., & Potthoff, S. (2006). Home telehealth improves clinical outcomes at lower cost for home healthcare. Telemedicine Journal and e-health: The official journal of the American Telemedicine Association*,* 12(2), 128–136. 10.1089/tmj.2006.12.128 16620167

[r17] First, M. B., Williams, J., Benjamin, L. S., & Spitzer, R. L. (2015). *User’s guide for the SCID-5-PD* (Structured Clinical Interview for DSM-5 Personality Disorder). American Psychiatric Association.

[r18] First, M. B., Williams, J. B., Karg, R. S., & Spitzer, R. L. (2016). *Structured Clinical Interview for DSM-5 Disorders, Clinician Version* (SCID-5-CV). American Psychiatric Association.

[r19] Foa, E. B., Ehlers, A., Clark, D. M., Tolin, D. F., & Orsillo, S. M. (1999). The Posttraumatic Cognitions Inventory (PTCI): Development and validation. Psychological Assessment, 11(3), 303–314. 10.1037/1040-3590.11.3.303

[r20] Kantor, V., Knefel, M., & Lueger-Schuster, B. (2017). Perceived barriers and facilitators of mental health service utilization in adult trauma survivors: A systematic review. Clinical Psychology Review, 52, 52–68. 10.1016/j.cpr.2016.12.001 28013081

[r21] Lewis, C., Roberts, N. P., Andrew, M., Starling, E., & Bisson, J. I. (2020). Psychological therapies for post-traumatic stress disorder in adults: Systematic review and meta-analysis. European Journal of Psychotraumatology, 11(1), 1729633. 10.1080/20008198.2020.1729633 32284821 PMC7144187

[r22] Meltzer, E. C., Averbuch, T., Samet, J. H., Saitz, R., Jabbar, K., Lloyd-Travaglini, C., & Liebschutz, J. M. (2012). Discrepancy in diagnosis and treatment of post-traumatic stress disorder (PTSD): Treatment for the wrong reason. The Journal of Behavioral Health Services & Research, 39(2), 190–201. 10.1007/s11414-011-9263-x 22076315 PMC3310322

[r23] Messman-Moore, T. L., & Bhuptani, P. H. (2017). A review of the long‐term impact of child maltreatment on posttraumatic stress disorder and its comorbidities: An emotion dysregulation perspective. Clinical Psychology: Science and Practice, 24(2), 154–169. 10.1111/cpsp.12193

[r24] Morina, N., Lancee, J., & Arntz, A. (2017). Imagery rescripting as a clinical intervention for aversive memories: A meta-analysis. Journal of Behavior Therapy and Experimental Psychiatry, 55, 6–15. 10.1016/j.jbtep.2016.11.003 27855298

[r25] Oprel, D. A. C., Hoeboer, C. M., Schoorl, M., de Kleine, R. A., Cloitre, M., Wigard, I. G., van Minnen, A., & van der Does, W. (2021). Effect of Prolonged Exposure, intensified Prolonged Exposure and STAIR+Prolonged Exposure in patients with PTSD related to childhood abuse: A randomized controlled trial. European Journal of Psychotraumatology, 12(1), 1851511. 10.1080/20008198.2020.1851511 34630934 PMC8500700

[r26] Paulik, G., Maloney, G., Arntz, A., Bachrach, N., Koppeschaar, A., & McEvoy, P. (2021). Delivering imagery rescripting via telehealth: Clinical concerns, benefits, and recommendations. Current Psychiatry Reports, 23(5), 24. 10.1007/s11920-021-01238-8 33725200 PMC7962431

[r27] Raabe, S., Ehring, T., Marquenie, L., Olff, M., & Kindt, M. (2015). Imagery rescripting as stand-alone treatment for posttraumatic stress disorder related to childhood abuse. Journal of Behavior Therapy and Experimental Psychiatry, 48, 170–176. 10.1016/j.jbtep.2015.03.013 25898289

[r28] Siegesleitner, M., Strohm, M., Wittekind, C. E., Ehring, T., & Kunze, A. E. (2019). Effects of imagery rescripting on consolidated memories of an aversive film. Journal of Behavior Therapy and Experimental Psychiatry, 62, 22–29. 10.1016/j.jbtep.2018.08.007 30176538

[r29] Simpson, S. (2009). Psychotherapy via videoconferencing: A review. British Journal of Guidance & Counselling, 37(3), 271–286. 10.1080/03069880902957007

[r30] Sunjaya, A. P., Chris, A., & Novianti, D. (2020). Efficacy, patient-doctor relationship, costs and benefits of utilizing telepsychiatry for the management of post-traumatic stress disorder (PTSD): A systematic review. Trends in Psychiatry and Psychotherapy, 42(1), 102–110. 10.1590/2237-6089-2019-0024 32321088

[r31] Üstün, T. B., Kostanjsek, N., Chatterji, S., & Rehm, J. (2010). *Measuring health and disability: Manual for WHO disability assessment schedule WHODAS 2.0.* World Health Organization. 10.2471/BLT.09.067231 PMC2971503 21076562

[r32] van den Berg, D. P., de Bont, P. A., van der Vleugel, B. M., de Roos, C., de Jongh, A., Van Minnen, A., & van der Gaag, M. (2015). Prolonged exposure vs eye movement desensitization and reprocessing vs waiting list for posttraumatic stress disorder in patients with a psychotic disorder: A randomized clinical trial. JAMA Psychiatry, 72(3), 259–267. 10.1001/jamapsychiatry.2014.2637 25607833

[r33] Varker, T., Brand, R. M., Ward, J., Terhaag, S., & Phelps, A. (2019). Efficacy of synchronous telepsychology interventions for people with anxiety, depression, posttraumatic stress disorder, and adjustment disorder: A rapid evidence assessment. Psychological Services, 16(4), 621–635. 10.1037/ser0000239 29809025

[r34] Weathers, F. W., Bovin, M. J., Lee, D. J., Sloan, D. M., Schnurr, P. P., Kaloupek, D. G., Keane, T. M., & Marx, B. P. (2018). The Clinician-Administered PTSD Scale for DSM-5 (CAPS-5): Development and initial psychometric evaluation in military veterans. Psychological Assessment, 30(3), 383–395. 10.1037/pas0000486 28493729 PMC5805662

[r35] Weathers, F. W., Litz, B. T., Keane, T. M., Palmieri, P. A., Marx, B. P., & Schnurr, P. P. (2013). *The PTSD Checklist for DSM-5 (PCL-5)**.* https://www.ptsd.va.gov

[r36] Wibbelink, C. J. M., Lee, C. W., Bachrach, N., Dominguez, S. K., Ehring, T., van Es, S. M., Fassbinder, E., Köhne, S., Mascini, M., Meewisse, M.-L., Menninga, S., Morina, N., Rameckers, S. A., Thomaes, K., Walton, C. J., Wigard, I. G., & Arntz, A. (2021). The effect of twice-weekly versus once-weekly sessions of either imagery rescripting or eye movement desensitization and reprocessing for adults with PTSD from childhood trauma (IREM-Freq): A study protocol for an international randomized clinical trial. Trials, 22(1), 848. 10.1186/s13063-021-05712-9 34838102 PMC8626728

[r37] Wild, J., Warnock-Parkes, E., Murray, H., Kerr, A., Thew, G., Grey, N., Clark, D. M., & Ehlers, A. (2020). Treating posttraumatic stress disorder remotely with cognitive therapy for PTSD. European Journal of Psychotraumatology, 11(1), 1785818. 10.1080/20008198.2020.1785818 33029325 PMC7473124

